# Giant Hypopharyngeal Fibrovascular Polyp: A Case Report and Review of the Relevant Literature

**DOI:** 10.1155/2015/670302

**Published:** 2015-11-30

**Authors:** Suheyl Haytoglu, Birgul Tuhanioglu, Abdurrahman Bozkurttan, Osman Kursat Arikan

**Affiliations:** Adana Numune Training and Research Hospital, ENT Clinics, 01260 Adana, Turkey

## Abstract

Fibrovascular polyps occur most commonly in the cervical esophagus and are extremely rare in the hypopharynx. In this paper, we report a case of fibrovascular polyp of a 52-year-old female, who presented with progressive dysphagia and weight loss and regurgitating a mass from her mouth. By the endoscopic examination, a polyp covered by normal mucosa with a wide stalk was detected at the hypopharynx. The pedicle of the mass was identified under general anesthesia and the 13 × 3 × 2 cm mass was completely resected perorally. Histopathological examination of the tumor showed oedematous subepithelial fibrous stroma, surrounded by squamous epithelium and containing many congested vascular structures. No recurrence was detected over one year of follow-up. This case highlights the need for clinicians to be aware of this rare entity and to develop the best approach to patient management.

## 1. Introduction

Fibrovascular polyp of the hypopharynx and esophagus, a rare, benign, intraluminal, submucosal tumor, is most commonly originated from proximal esophagus [1, 2]. This lesion, usually unique, affects predominantly men with an average age of 53 years [3, 4]. Fibrovascular polyps are usually asymptomatic and small, detected by endoscopy. They can grow to considerable length and cause digestive or respiratory symptoms [4]. These tumors can attain very large sizes after slow growth over a long period [5].

The most common complaints include dysphagia and a sensation of a mass. Other complaints are retrosternal or epigastric discomfort, odynophagia, vomiting, weight loss, and respiratory symptoms such as persisting cough and shortness of breath. The most distinctive feature of a fibrovascular polyp is regurgitation into the mouth [3]. The asphyxia and laryngeal obstruction of the polyp regurgitation may cause sudden death which is the most feared complication [6, 7]. When the polyp twists, it leads to hemorrhage and necrosis of the lesion [8]. Surgical removal of fibrovascular polyp is usually suggested.

## 2. Case Report

**Figure 1:**
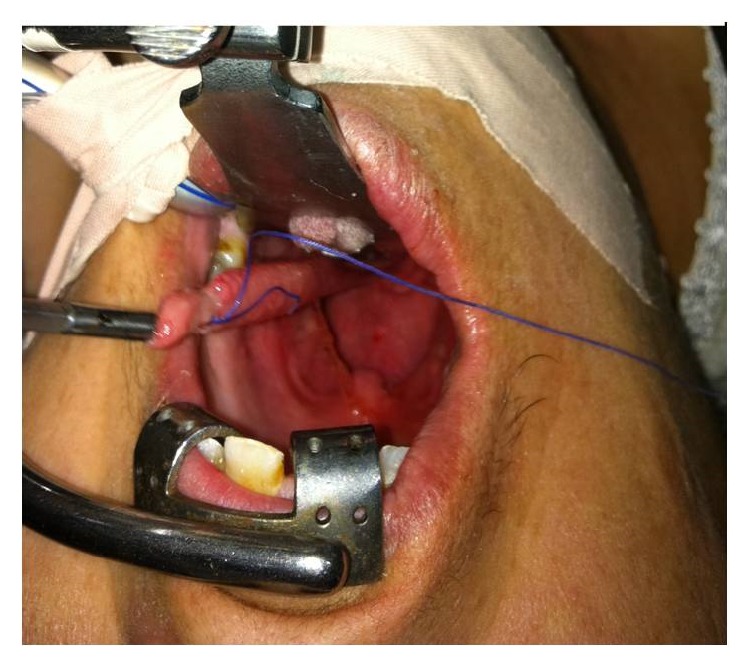
View of giant fibrovascular polyp originating from hypopharynx.

**Figure 2:**
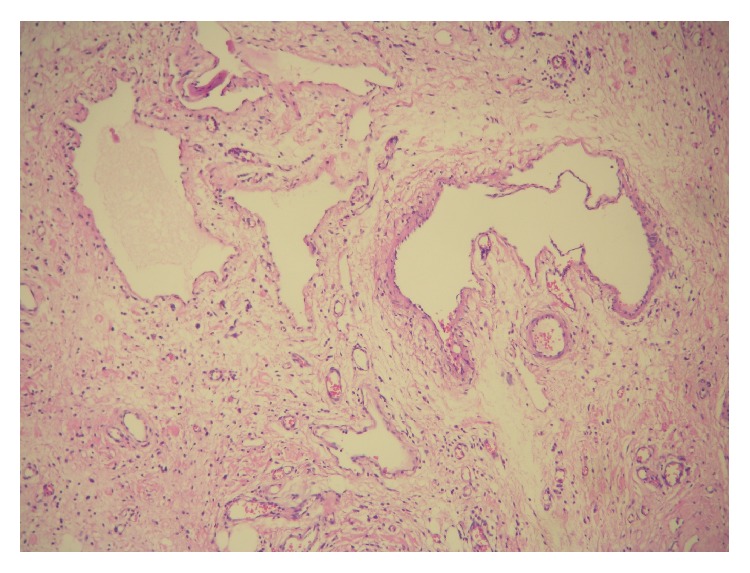
Microscopic view showed an oedematous subepithelial fibrous stroma, surrounded by squamous epithelium and containing many congested vascular structures (100x, HE).

A 52-year-old woman presented to the gastroenterology department with complaints of progressive dysphagia, and regurgitating a mass from her mouth. The patient suffered from this complaint for 1 year. She also suffered from losing weight. Her past medical history was not significant except for diabetes mellitus of 2 years. The patient underwent endoscopy in the gastroenterology department. The esophagogastroscopy demonstrated a 50 mm sized polyp with stalk at the hypopharynx. The patient was referred to the Otolaryngology Department of Adana Numune Training and Research Hospital with the impression of polyp of the hypopharynx. The endoscopic examination showed a mass with wide pedicle covered with smooth mucosa at the hypopharynx. An operation was planned. The laryngoscopic and esophagoscopic evaluation of the hypopharynx under general anesthesia revealed that the stalk was attached to the posterior wall of the hypopharynx (Figure 1). The pedicle of the mass was identified and the 13 × 3 × 2 cm mass was completely resected by electrocautery perorally. Histopathological examination of the tumor showed an oedematous subepithelial fibrous stroma, surrounded by squamous epithelium and containing many congested vascular structures (Figure 2). No recurrence was detected over one year of follow-up.

The patient has been informed and the approval form has been signed by the patient to publish her medical data for scientific publications and presentations.

## 3. Discussion

Benign hypopharyngeal tumors are uncommon and their exact incidence is unknown [9]. Fibrovascular polyps of the esophagus and hypopharynx are benign tumors of the upper digestive tract. Malignant transformation of these tumors is reportedly rare [10].

It is reported that sarcomas can develop from the lipomatous component of the esophageal polyp; otherwise, the squamous carcinomas can develop from the mucosa of the polyp separately [11].

Fibrovascular polyps usually originate as small mucosal tumors; then, they grow up by the pressure changes with the constant downward urge of both food and peristalsis [2]. Fibrovascular polyps of hypopharynx occur commonly in older men between 60 and 70 years of age [2]. In our case, the patient was a 52-year-old woman.

Fibrovascular polyp may remain asymptomatic for long years because of its slow-growing nature. In the literature, dysphagia is the most common complaint. Other complaints are sensation of a mass, retrosternal discomfort, epigastric pain, regurgitating the polyp into the mouth, loss of weight, and persistent cough [7, 12]. In our case, the patient presented with progressive dysphagia and regurgitating a mass in her mouth. In the literature, life-threatening symptoms as asphyxia and laryngeal obstruction by the polyp were reported [6, 7]. The diagnosis of a fibrovascular polyp of the hypopharynx is difficult if the mass is smaller than 2 cm in diameter. The endoscopy may not detect the small polyp because it is an intramural mass covered by normal mucosa of the hypopharynx. In our case, the polyp was about 13 × 3 × 2 cm and it could be diagnosed by endoscopy. However, Whitman and Borkowski have defined the computed tomography and magnetic resonance imaging findings for fibrovascular polyps; the radiological study is not required for our patient because the stalk of the polyp was visualized completely by the endoscopic evaluation [13].

Because of its progressive nature and the risk of sudden death by asphyxia, surgical removal of the polyp is recommended and local excision of the polyp is curative; the recurrence is rare [9].

The surgical technique is chosen after assessing the origin, size, and vascularity of the pedicle [2]. Small polyps less than 2 cm in diameter with a thin pedicle can be treated by endoscopic resection. Pham et al. described the endoscopic removal of a giant fibrovascular polyp in the esophagus [14].

If the stalk of the polyp can be visualized completely, peroral excision with the assistance of electrocautery is also an option. For larger polyps, Owens et al. reported two patients operated on by lateral pharyngotomy [10].

Hoseok et al. reported performing a biapproach surgical treatment (transcervical and transabdominal approach simultaneously) for excision of a giant fibrovascular polyp of the upper digestive tract [15].

In our case, we performed a peroral excision with the assistance of electrocautery. The bipolar cautery was used for controlling hemorrhage. We had no complication and bleeding problem during the operation. The patient has been followed up without any recurrence for 1 year postoperatively.

## 4. Conclusion

If the patient presents with slow growing, progressive dysphagia, endoscopic evaluation is needed. Although the hypopharyngeal and esophageal tumors are mostly malign, the specialist must bear in mind that the tumor could be benign. Fibrovascular polyps should be considered in the differential diagnosis of the tumors in this area. Excision the fibrovascular polyp is curative and the recurrence risk is rare. If the stalk of the fibrovascular polyp is completely in view, there is no need to perform an open surgery because of the increased morbidity.
